# BDNF Protein and BDNF mRNA Expression of the Medial Prefrontal Cortex, Amygdala, and Hippocampus during Situational Reminder in the PTSD Animal Model

**DOI:** 10.1155/2021/6657716

**Published:** 2021-03-08

**Authors:** Shao-Han Chang, Ying Hao Yu, Alan He, Chen Yin Ou, Bai Chuang Shyu, Andrew Chih Wei Huang

**Affiliations:** ^1^Taiwan International Graduate Program in Interdisciplinary Neuroscience, National Cheng Kung University and Academia Sinica, Taipei, Taiwan; ^2^Institute of Biomedical Sciences, Academia Sinica, Taipei, Taiwan; ^3^Department of Psychology, Fo Guang University, Yilan County 26247, Taiwan; ^4^Department of Biotechnology and Animal Science, National Ilan University, Yilan 26047, Taiwan

## Abstract

Whether BDNF protein and BDNF mRNA expression of the medial prefrontal cortex (mPFC; cingulated cortex area 1 (Cg1), prelimbic cortex (PrL), and infralimbic cortex (IL)), amygdala, and hippocampus (CA1, CA2, CA3, and dentate gyrus (DG)) was involved in fear of posttraumatic stress disorder (PTSD) during the situational reminder of traumatic memory remains uncertain. Footshock rats experienced an inescapable footshock (3 mA, 10 s), and later we have measured fear behavior for 2 min in the footshock environment on the situational reminder phase. In the final retrieval of situational reminder, BDNF protein and mRNA levels were measured. The results showed that higher BDNF expression occurred in the Cg1, PrL, and amygdala. Lower BDNF expression occurred in the IL, CA1, CA2, CA3, and DG. BDNF mRNA levels were higher in the mPFC and amygdala but lower in the hippocampus. The neural connection analysis showed that BDNF protein and BDNF mRNA exhibited weak connections among the mPFC, amygdala, and hippocampus during situational reminders. The present data did not support the previous viewpoint in neuroimaging research that the mPFC and hippocampus revealed hypoactivity and the amygdala exhibited hyperactivity for PTSD symptoms. These findings should be discussed with the previous evidence and provide clinical implications for PTSD.

## 1. Introduction

Posttraumatic stress disorder (PTSD) is a severe and chronic mental illness. PTSD symptoms can be caused by severe traumatic events, including illness (e.g., cancer [1, 2]), situations of conflict (e.g., war [3]), and natural disasters (e.g., earthquakes [4]). According to the *Diagnostic and Statistical Manual of Mental Disorders* (DSM-5), PTSD has numerous critical symptoms [5]. For example, patients may persistently suppress stimuli associated with the traumatic stimulus and induce emotional numbing [6] by reexposing the environmental stimulus (i.e., the conditioned stimulus (CS)) associated with previous traumatic events (i.e., the unconditioned stimulus (US)) [7]. Patients with PTSD often experience persistent traumatic events as well as feelings of fear, helplessness, and horror [8, 9]. In the animal model of PTSD, growing studies employed the procedure of the situational reminder to imitate PTSD patients who continuously experience traumatic events [10–14]. Therefore, the present study used the procedure of situational reminder to test fear for PTSD symptoms.

Brain-derived neurotrophic factor (BDNF) is a signal that regulates axon and dendrite growth [15]. The intracellular signaling cascade of BDNF is associated with tropomyosin-related kinase B (TrkB) receptors to govern neuronal survival, axonal growth, and synaptic plasticity [16, 17]. Previous studies have reported that BDNF expression in the medial prefrontal cortex (mPFC), amygdala, and hippocampus was likely associated with stress-related events and PTSD symptoms [16]. For example, the early-weaned mice showed increased freezing behavior following fear conditioning, and these mice exhibited decreases in BDNF expression and mRNA transcripts for BDNF exon III in the mPFC [18]. Using the single prolonged-stress footshock procedure of the PTSD model indicated that the prefrontal cortex has lower BDNF levels [19]. BDNF secretions in the ventral hippocampus-infralimbic cortex (IL) pathway could alter the fear contextual conditioning [20]. Under long-term restraint stress, the basolateral amygdala (BLA) exhibited higher levels in BDNF protein and BDNF mRNA expression; moreover, the CA3 of the hippocampus has lower BDNF protein and BDNF mRNA expression [21]. A study of predator scent stress in animals suggested that neuropeptide S microinjections in the BLA reduced stress-related behavior and ameliorated low levels of BDNF in the BLA [22]. As animals with early footshock experiences and then receiving a cue fear conditioning, the footshock decreased BDNF expression in the dentate gyrus (DG) of the hippocampus in the PTSD animal model [23]. Recently, the inhibition of the hyperpolarization-activated cyclic nucleotide-gated channel 1 (HCN1) was revealed to reduce stress-related immobility behavior and escaped time in the water maze test; moreover, BDNF-mTOR signaling in the prefrontal cortex and the hippocampus was facilitated by the inhibition of the HCN1 [24]. Sleep deprivation after contextual conditioning was shown to reduce BNDF and p-ERK levels in the hippocampus and amygdala and attenuated memory retrieval [25]. Nevertheless, fewer studies provided conflict data related to the BDNF involvements of the mPFC, amygdala, and hippocampus in stress or PTSD [26, 27]. For example, chronic stress treatments were negatively associated with BDNF mRNA expression and positively linked with *TrkB* mRNA expression in the CA1 of the hippocampus [26]. Recently, a research study found that acute treatment with ketamine reduced freezing behavior, but this treatment did not affect BDNF expression or glucose metabolism in the hippocampus, frontal cortex, or amygdala in the PTSD animal model [27]. Therefore, whether BDNF expression of the frontal cortex, amygdala, and hippocampus regulated PTSD symptoms should be scrutinized in the present study.

On the other hand, the review paper suggested that the mPFC as well as the hippocampus was negatively connected to the amygdala; moreover, the mPFC was positively connected with the hippocampus [28]. In this study, the mPFC and hippocampus exhibited lower neural activity and the amygdala appeared to have higher neural activity when PTSD patients suffered from a trauma event [28]. However, these data were examined in the neuroimaging research but not in the other approaches. Therefore, the present study used the labeling approach of BDNF protein and BDNF mRNA to reexamine how the contribution of the neural connections among the mPFC, amygdala, and hippocampus regulated fear behavior of PTSD symptoms, especially for the situational reminder phase.

To target these emerged issues, this study concerned whether a footshock-induced severe traumatic memory event produced a fear response in the animal model of PTSD during the situational reminder phase. Moreover, the present work examined whether the mPFC (e.g., Cg1, PrL, and IL), hippocampus (e.g., CA1, CA2, CA3, and DG), amygdala, and piriform cortex (PC) exhibited higher BDNF protein and BDNF mRNA expression for PTSD-like rats, and it also tested the connections among the mPFC (i.e., Cg1, PrL, and IL), hippocampus (i.e., CA1, CA2, CA3, and DG), amygdala, and PC by analyzing the data of BDNF or BDNF mRNA in the third retrieval session of situational reminder.

## 2. Experimental Procedure

### 2.1. Animals

Forty-six male Wistar rats were purchased from BioLASCO Taiwan Co., Ltd. (Yilan County, Taiwan). At the beginning of the experiments, the weight of each rat was 250–350 g. All the rats were group-housed, two per plastic cage, with wooden bedding in the cage. The cages were kept in a colony room with a constant temperature (approximately 23 ± 2°C) and light phase between 6:00 a.m. and 6:00 p.m. Food and water were provided *ad libitum*. The experiments were carried out in compliance with the American Psychological Association ethical standards for the treatment of animals. A description of the details of the treatment was submitted and received approval (ethical protocol # 1080008) from the Institutional Animal Care and Use Committee (IACUC) of Fo Guang University. Every effort was made to minimize the animals' suffering and the number of animals used.

### 2.2. Apparatus

The inescapable footshock apparatus is a box with a surrounding plastic shell measuring 60 cm × 60 cm × 72 cm high. The floor of the apparatus comprises metal grids (0.3 cm diameter at 0.7 cm grid intervals).

### 2.3. Behavioral Procedure

**Figure 1:**
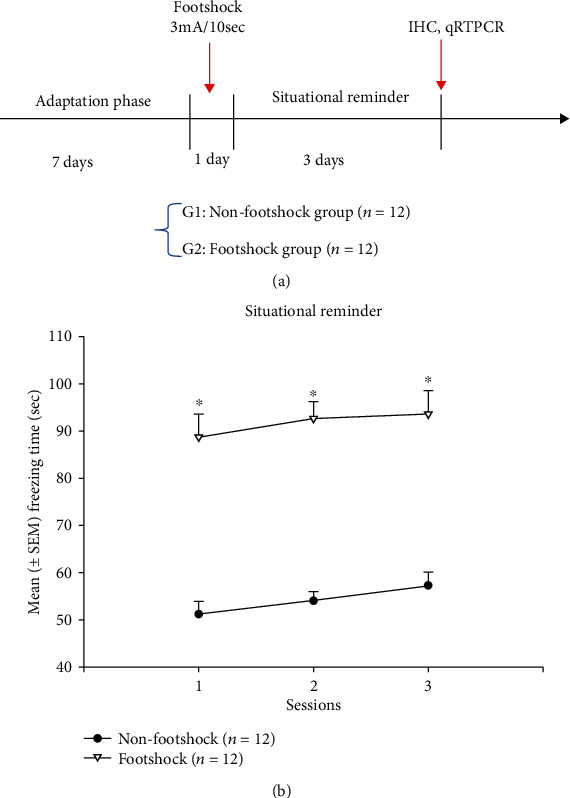
(a) Schematic representation of the experiment paradigm. After the seven-day adaptation phase, 3 mA footshock for 10 seconds was applied for fear conditioning, and freezing levels were measured for three sessions during situational reminder. Two hours after the third retrieval session of freezing behavior measurement, the rats were euthanized and their brain tissues were collected and further processed for immunohistochemical staining and qRT-PCR analysis. (b) Mean (±SEM) freezing time for three sessions during situational reminder. Conditioned freezing behavior was measured in the nonfootshock (*n* = 12) and footshock (*n* = 12) groups. ^∗^*p* < 0.05 when comparing the nonfootshock and footshock groups.

The experimental procedure is shown in Figure 1(a). Following the seven-day adaptation phase was the conditioning phase, where the rats were divided into nonfootshock (control group, *n* = 12) and footshock (*n* = 12) groups. During this phase, the rats in the footshock group received an inescapable footshock (3 mA, 10 seconds) and were then kept for two minutes in the footshock box. The rats in the control group were placed in the chamber for an equivalent period without receiving a footshock. Following this, rats were reexposed to the footshock box for two minutes to induce situational reminders once a day for three days. The experimental procedure of the PTSD animal model in situational reminder was referred from the previous studies [10, 14].

To examine BDNF proteins in the selected brain areas, immunohistochemical (IHC) staining was conducted on the nonfootshock (*n* = 4) and footshock (*n* = 6) groups following the third session of the situational reminder phase. The quantitative real-time polymerase chain reaction (qRT-PCR) method was performed on the nonfootshock (*n* = 6) and footshock (*n* = 6) groups to measure BDNF mRNA expression. Because fear behavior is a crucial PTSD symptom, the study addressed whether such behavior occurred in the third retrieval session. Thus, in the third retrieval session, qRT-PCR was performed to label BDNF proteins and BDNF mRNA levels.

The animals were euthanized, and their brain tissues were collected for further analysis of BDNF expression by IHC staining or BDNF mRNA levels. BDNF-positive nuclei were determined in conditioned fear-associated brain regions, including the Cg1, PrL, IL, hippocampus (CA1, CA2, CA3, and DG), amygdala, and PC. BDNF mRNA levels were labeled in the mPFC, hippocampus, amygdala, and PC.

### 2.4. Behavioral Testing

Situational reminders were conducted to reexperience the PTSD trauma event by measuring freezing behavior in rats, which was video recorded for two minutes in the previous environment which was associated with footshock. Freezing behavior comprises an index of fear responses, defined as the absence of all movements except respiration [29].

### 2.5. Immunohistochemical Staining of BDNF

Based on the previous findings related to the time course of BDNF protein expression [30], the immunohistochemical staining with BDNF was performed 60~90 minutes after the final session of freezing behavior measurement. The rats were euthanized with an overdose of sodium pentobarbital. Then, the rats were perfused with 0.9% sodium chloride followed by 4% paraformaldehyde in 0.1 M phosphate-buffered saline (PBS). After perfusion, the brain tissues were removed and postfixed in 4% paraformaldehyde for three days. Following this, the brain tissues were stored in 30% sucrose until the brain sank. Later, a brain microdissection procedure was performed. The brain tissue was embedded by using frozen gel (Tissue-Tek O.C.T. compound) before sectioning. The frozen brain was placed in the platform of the microtome, and during the brain microdissection, coronal sections were cut 40 *μ*m thick in a freezing microtome chamber. The temperature in the microtome chamber was maintained at -20°C. The coronal sections of the brain were collected in 0.1 M PBS. Anterior and posterior coordinates were by the brain map of rats [31].

Alternate sections were picked, and free-floating sections were washed once for 10 minutes in 0.1 M PBS and then immersed in 3% hydrogen peroxide (H_2_0_2_) to block endogenous peroxidase and 1% Triton X-100 to enhance membrane permeability. After rinsing in 0.1 M PBS, the brain slices were submerged in normal goat serum with 0.1% Triton (NGST) for one hour and incubated overnight at 4°C with an anti-BDNF antibody (Millipore/AB1513, 1 : 500). The following day, the brain slices were rinsed for 10 minutes in 0.1 M PBS and incubated in a secondary biotinylated rabbit anti-sheep IgG antibody (1 : 500, BA-6000, Vector Laboratories, CA, USA) in 1% NGST at room temperature for one hour. The slices were then rinsed again in 0.1 M PBS for 10 minutes, and the bound secondary antibody was placed in an avidin-biotin solution in 0.1 M PBS (ABC kit, Vector Laboratories, CA, USA) for one hour. After this, the slices were rinsed once again in 0.1 M PBS for 10 minutes and then incubated with a chromogen reaction solution (PBS, pH 7.4, 3% H_2_O_2_, 25% nickel, and 0.03% 3,3′-diaminobenzidine) for 10 minutes. Finally, all sections were rinsed in a PBS solution and mounted onto gelatin-coated slides. For quantifying BDNF expression, nuclei with positive dark-point immunoreactivity were counted visually at 20x magnification. The counting software ImageJ was applied to count the c-Fos-positive neurons. The counts of the slices for each brain subarea were averaged for each group.

### 2.6. Real-Time Quantitative PCR of BDNF

Total RNA was extracted using the TRIzol reagent (Invitrogen, Carlsbad, CA, USA) according to the manufacturer's instructions. Total RNA was used for cDNA synthesis with random hexamers. The next step was the reverse transcription PCR amplification of BDNF. The amplification was initiated with a pair of BDNF primers (forward: 5′-AAAACCATAAGGACGCGGACTT-3′; reverse: 5′-AAAGAGCAGAGGAGGCTCCAA-3′) in a total reaction volume of 20 *μ*l, undergoing a denaturation stage at 95°C for 10 minutes, followed by 28 cycles of denaturation at 95°C for one minute, primer annealing at 55°C for 30 seconds, and extension at 72°C for 45 seconds. Once the cycling steps were complete, the final extension was at 72°C for five minutes. The reactions were repeated three times and were performed in an ABI PRISM 7500 Sequence Detection System (Applied Biosystems, Thermo Fisher Scientific, USA). The mean expression level of the housekeeping gene with a pair of beta-actin primers (forward: 5′CAACTTGATGTATGAAGGCTTTGGT-3′; reverse: 5′-ACTTTTATTGGTCTCAAGTCAGTGTACAG-3′) was used as the internal control to normalize the variability of BDNF expression levels. The relative changes in gene expression were analyzed using the 2^−ΔΔCT^ method, as described in a previous study [32].

### 2.7. Statistical Analysis

A two-way mixed (group vs. session) analysis of variance (ANOVA) was performed for the freezing time. BDNF expression and normalized BDNF mRNA were analyzed using an independent *t*-test for a specific brain area of the nonfootshock and footshock groups. To examine the relationship between freezing response and the BNDF expressions or freezing response and BDNF mRNA, Pearson correlation tests were conducted. Values of *p* < 0.05 were considered to be statistically significant. The heat map of the neural networks in the determined brain areas was transformed from Pearson correlation coefficient values and illustrated by MATLAB free packages (The MathWorks, Inc., Natick, MA, USA). Note that the higher value of the Pearson correlation coefficient indicated a long-wave color. The lower value of the Pearson correlation coefficient showed a short-wave color. The values of power were analyzed following Pearson correlation tests.

## 3. Results

### 3.1. Freezing Behavior Tests during the Situational Reminder

In this study, a single severe footshock was paired with the context of the footshock box. Then, the animals encountered an experimental procedure of situational reminders in the footshock box. In this procedure, animals were given without any footshock once a day for three days to mimic patients with PTSD who have reexperienced a traumatic memory. By testing freezing behavior during situational reminder, a two-way mixed ANOVA (footshock vs. session) indicated that a significant difference occurred in the group (*F*_1,22_ = 98.00, *p* < 0.05; partial eta square = 0.82, power = 1.00). Nonsignificant differences occurred in session (*F*_2,44_ = 1.62, *p* > 0.05; partial eta square = 0.07, power = 0.32) and in the interaction of the group and session (*F*_2,44_ = 0.07, *p* > 0.05; partial eta square = 0.003, power = 0.06). The results highlight that footshock treatments significantly increased freezing time compared to the nonfootshock group for three sessions during situational reminder (Figure 1(b)).

### 3.2. BDNF Immunohistochemical Staining during Situational Reminder and Fear Behavior in PTSD-Associated Brain Areas

**Figure 2:**
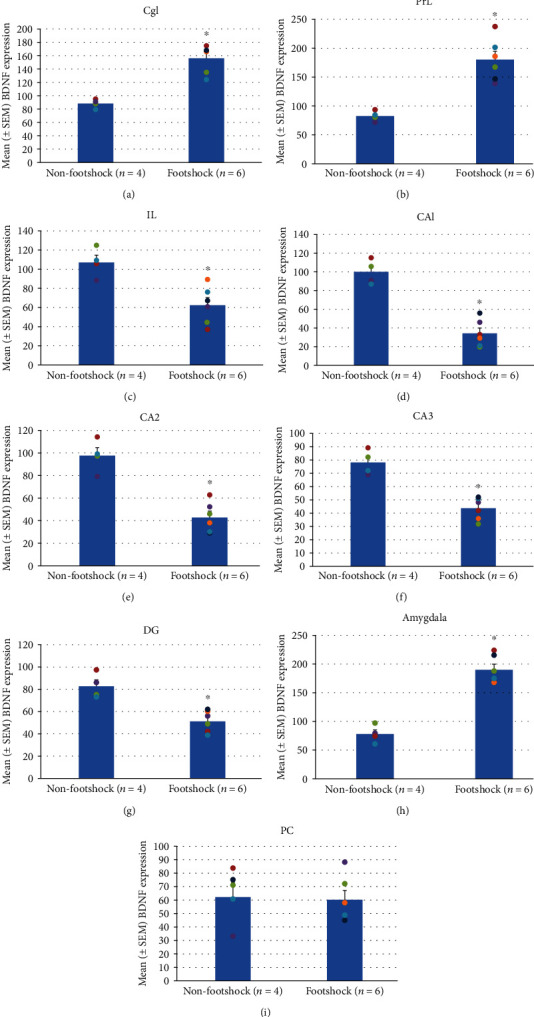
(a–i) Mean (±SEM) BDNF-positive cells per slice in footshock (*n* = 4) and nonfootshock (*n* = 6) groups. The number of BDNF-positive cells was counted in the PTSD-associated regions, including the Cg1; PrL; IL; hippocampal areas CA1, CA2, CA3, and DG; amygdala; and PC. ^∗^*p* < 0.05 when comparing the nonfootshock and footshock groups.

**Figure 3:**
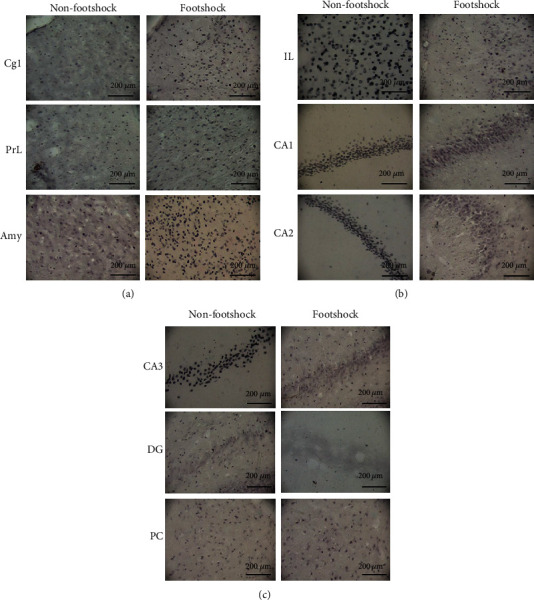
(a–c) Representative photomicrographs of BDNF-positive cells in the region of the Cg1; PrL; IL; hippocampal areas CA1, CA2, CA3, and DG; amygdala; and PC.

By investigating the involvement of brain areas in the BDNF expression of the nonfootshock and footshock groups, an independent *t*-test indicated that the footshock group experienced significant increases in BDNF expression in the Cg1 (*t*(8) = −6.17, *p* < 0.05; Figure 2(a)), PrL (*t*(8) = −5.07, *p* < 0.05; Figure 2(b)), and amygdala (*t*(8) = −8.17, *p* < 0.05; Figure 2(h)). In contrast, the BDNF expression of the footshock group in the IL (*t*(8) = 3.86, *p* < 0.05; Figure 2(c)), CA1 (*t*(8) = 7.35, *p* < 0.05; Figure 2(d)), CA2 (*t*(8) = 6.14, *p* < 0.05; Figure 2(e)), CA3 (*t*(8) = 6.21, *p* < 0.05; Figure 2(f)), and DG (*t*(8) = 4.80, *p* < 0.05; Figure 2(g)) showed a significant decrease compared to that of the nonfootshock group. The BDNF expression of the PC between the nonfootshock and footshock groups had no significant differences (*t*(8) = 0.17, *p* > 0.05; Figure 2(i)). To compare both of these groups, the BDNF expression of the determined brain areas is shown in Figures 3(a)–3(c). These results suggest that the Cg1, PrL, IL, CA1, CA2, CA3, DG, and amygdala were shown to have higher BDNF protein expression in the PTSD during situational reminder.

### 3.3. Quantification of BDNF mRNA Levels during Situational Reminder and Fear Behavior in PTSD-Associated Brain Areas

**Figure 4:**
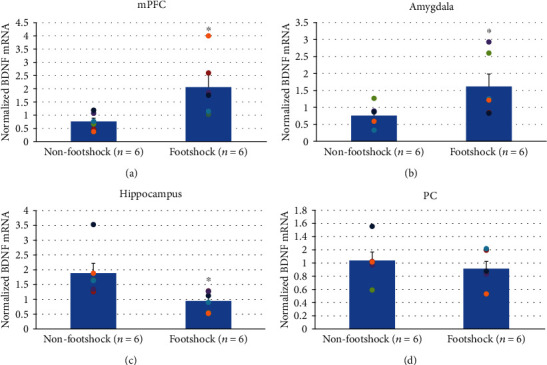
Normalized BDNF mRNA in the mPFC, amygdala, hippocampus, and PC in footshock (*n* = 6) and nonfootshock (*n* = 6) groups. ^∗^*p* < 0.05 when comparing the nonfootshock and footshock groups.

The BDNF mRNA levels were further determined by qRT-PCR. An independent *t*-test indicated that significantly higher BDNF mRNA levels occurred in the mPFC in the footshock group (*t*(10) = −2.79, *p* < 0.05; Figure 4(a)) and amygdala (*t*(10) = −2.16, *p* = 0.05; Figure 4(b)). The hippocampus showed significantly decreased BDNF mRNA levels in the footshock group (*t*(10) = 2.54, *p* < 0.05; Figure 4(c)). A nonsignificant difference occurred in the PC (*t*(10) = 0.69, *p* > 0.05; Figure 4(d)). The mPFC, amygdala, and hippocampus therefore mediate the BDNF mRNA levels.

### 3.4. Pearson Correlation Tests for Freezing Behavior and BDNF Protein Expressions in the Third Session of Situational Reminder

Rats induced freezing behavior following severe footshock treatment and were then placed in the same footshock box to induce fear behavior for three sessions during situational reminder. To examine the relationship between footshock-induced freezing behavior as a PTSD symptom and BDNF protein expressions in the brain, we assessed the relationship between freezing levels and BDNF protein expressions using Pearson correlation tests. The results indicated that freezing levels were positively associated with BDNF protein levels in the Cg1 (*r* = 0.72, *p* < 0.05), PrL (*r* = 0.76, *p* < 0.05), and amygdala (*r* = 0.75, *p* < 0.05). The associations in the subregions of the hippocampus were negatively correlated, including those in the CA1 (*r* = −0.76, *p* < 0.05), CA2 (*r* = −0.75, *p* < 0.05), and CA3 (*r* = −0.83, *p* < 0.05). However, freezing levels and BDNF protein expressions were not significantly correlated in the IL (*r* = −0.61, *p* > 0.05), DG (*r* = −0.60, *p* > 0.05), or PC (*r* = −0.49, *p* > 0.05; Table 1). Therefore, from the analysis of the relationship between freezing behavior and BDNF protein expressions, a positive correlation occurred in the Cg1, PrL, and amygdala; however, a negative correlation was found in the CA1, CA2, CA3, and amygdala.

### 3.5. Pearson Correlation Tests for Freezing Behavior and BDNF mRNA Levels in the Third Session of Situational Reminder

We also tested the relationship between freezing behavior and BDNF mRNA levels using Pearson correlation tests. It was found that a positively significant correlation occurred in the mPFC (*r* = 0.80, *p* < 0.05) and amygdala (*r* = 0.84, *p* < 0.05). A negative correlation was noted in the hippocampus (*r* = −0.62, *p* < 0.05). A nonsignificant correlation occurred in the PC (*r* = −0.28, *p* > 0.05; Table 2). Therefore, from the analysis of the relationship between freezing behavior and BDNF mRNA levels, positive correlations were observed in the mPFC and amygdala; however, a negative correlation occurred in the hippocampus.

### 3.6. Neural Connection Analysis of Regional BDNF Protein Expression

**Figure 5:**
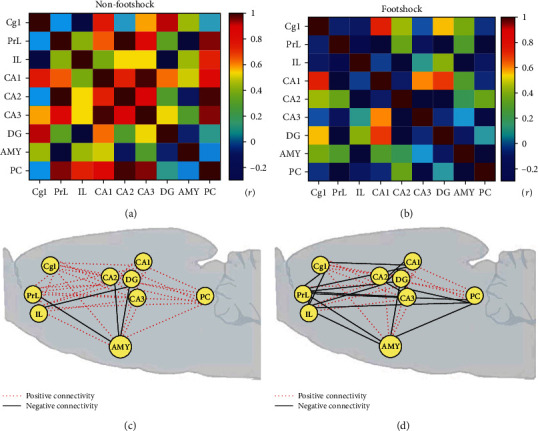
Pearson's correlation coefficient of BDNF protein expression in (a) nonfootshock (*n* = 4) and (b) footshock (*n* = 6) and the connectivity in (c) nonfootshock (*n* = 4) and (d) footshock (*n* = 6) between regions, including the Cg1; PrL; IL; hippocampal areas CA1, CA2, CA3, and DG; amygdala; and PC. Note that the red dotted line represents positive connectivity. The black line represents negative connectivity.

To test the neural connection of BDNF protein expression, we used a Pearson correlation analysis. For the nonfootshock and footshock groups, the values of Pearson correlation tests were shown to be 0.991~0.097 and -0.740~0.006, respectively. Moreover, the power values were 0.940~0.051 for the nonfootshock group and 0.436~0.050 for the footshock group. Thus, Pearson correlation coefficients were transferred to a heat map with colors to examine the relationship among the selected brain areas in BDNF expression. BDNF protein-level associations were analyzed in both nonfootshock (Figure 5(a)) and footshock (Figure 5(b)) groups. The heat map of the nonfootshock group exhibited many yellow and red colors, thus showing a higher correlation than any other comparison of brain areas (Figure 5(a)). However, after the footshock treatments, comparisons of all brain areas appeared to show more blue colors in the heat map, indicating that a footshock-induced traumatic event disrupted the relationship between all brain areas (Figure 5(b)). It was suggested that lower BDNF correlation levels were found in the brain following PTSD, thus representing a negatively regional connection in the third retrieval session of situational reminder. Furthermore, the neural connections during the third retrieval session of situational reminder for all neural substrates showed that a large amount of positive connectivity and a small amount of negative connectivity in nonfootshock (Figure 5(c)) become a lot of negative connectivity and less positive connectivity in BDNF expression in footshock (Figure 5(d)).

### 3.7. Neural Connection Analysis of Regional BDNF mRNA Levels

**Figure 6:**
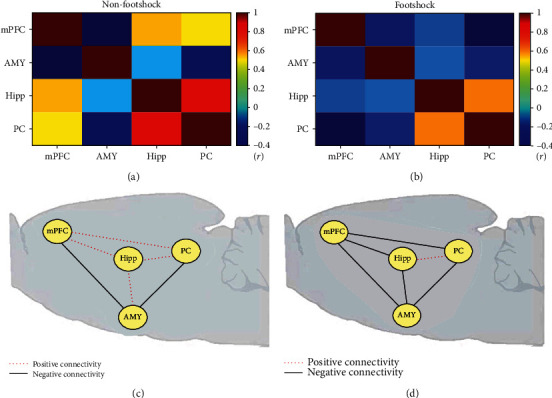
Pearson's correlation coefficient of BDNF mRNA levels in (a) nonfootshock (*n* = 6) and (b) footshock (*n* = 6) and the connectivity in (c) nonfootshock (*n* = 6) and (d) footshock (*n* = 6) between regions, including the Cg1; PrL; IL; hippocampal areas CA1, CA2, CA3, and DG; amygdala; and PC. Note that the red dotted line represents positive connectivity. The black line represents negative connectivity.

To test the neural connection of BDNF mRNA levels, we used a Pearson correlation analysis. For the nonfootshock and footshock groups, the values of Pearson correlation tests were shown to be 0.783~0.135 and -0.547~0.116, respectively. Moreover, the power values were 0.519~0.057 for the nonfootshock group and 0.203~0.055 for the footshock group. The value of the Pearson correlation coefficient was transferred into the heat map with color to examine the relationship among the mPFC, amygdala, hippocampus, and PC in BDNF mRNA levels for both nonfootshock and footshock groups (Figure 6). During the third retrieval session of situational reminder, the nonfootshock group revealed to have higher correlation values for the mPFC with the hippocampus and PC, the mPFC and hippocampus, and the mPFC and PC (Figure 6(a)); however, the Pearson correlation coefficients decreased for almost all comparisons for all determined brain areas in the footshock group (Figure 6(b)). Therefore, it appears that PTSD in situational reminders interferes with connections between the mPFC, amygdala, hippocampus, and PC in the brain, and it induces lower BDNF mRNA levels in footshock. It indicated that when compared with the nonfootshock and footshock groups, the connections of the neural network for all determined brain areas changed from having increased positive connectivity to decreased positive connectivity and enhanced negative connectivity in BDNF mRNA levels, especially in the situational reminder phase (Figures 6(c) and 6(d)).

## 4. Discussion

The present study results showed that the footshock rats still induced a severe freezing behavior in the third retrieval session of situational reminder and the behavioral data were consistent with the previous evidence [10, 13, 14]. The mPFC (i.e., Cg1 and PrL) and the amygdala appeared to have higher BDNF protein expression. In contrast, part of the mPFC (i.e., IL) and the hippocampus (i.e., CA1, CA2, CA3, and DG) showed lower BDNF protein expression in the third retrieval session of situational reminder. In the BDNF mRNA levels, the results were very similar to those in BDNF protein expression. The levels of BDNF mRNA were higher in the mPFC and amygdala but lower in the hippocampus for the footshock group in the third retrieval session of situational reminder.

The neural connection analysis suggested that some connections between the mPFC, amygdala, and hippocampus changed the connection property from positive to negative. These connections included the PrL projections to the subareas of the mPFC (i.e., PrL-Cg1 and PrL-IL) and the subareas of the hippocampus (i.e., PrL-CA2, PrL-DG, and PrL-CA3), the amygdala projections (i.e., IL-amygdala, amygdala-CA2, and amygdala-PC), and the projections of the IL-PC, PC-CA3, and CA1-CA2. Therefore, the situational reminder of traumatic memory weakened the neural network of the mPFC, amygdala, and hippocampus that exhibited negative connections.

### 4.1. The mPFC and PTSD

A growing body of evidence has shown that the subregions of the mPFC (such as the PrL, IL, and Cg1) play separate roles in fear conditioning and PTSD symptoms [33]. Previous studies have reported some controversial evidence in this regard [20, 34–37]. For example, a study of fear conditioning discrimination showed that the subdivisions of the mPFC activated different responses to fear discrimination learning, and the PrL and IL seemingly contributed counterbalanced roles in fear discrimination learning [34]. A previous study has reported that animals with a single prolonged-stress PTSD procedure revealed to have significantly lower BDNF expression in the hippocampus and mPFC and decreased phosphorylated TrkB receptors in the ventral mPFC. However, microinfusions of BDNF in the IL (but not in the PrL or hippocampus) reduced the impairment of fear extinction but not extinction training. BDNF microinjections in the IL significantly activated TrkB phosphorylation in the IL, indicating that the signaling of BDNF to TrkB receptors in the IL (but not the PrL and hippocampus) regulates fear extinction memory [35]. Using the resting-state method of functional magnetic resonance imaging, a study examined brain mechanisms of PTSD and found that PTSD patients appeared to have a higher level of functional connectivity in the left posterior hippocampus and bilateral posterior cingulate cortex compared to the trauma-exposed control group, indicating that the cingulate cortex of the mPFC may be involved in PTSD fear behavior [36].

The present study focused on the situational reminder of traumatic memory, and the Cg1 and PrL of the mPFC showed higher BDNF expression; however, the IL exhibited lower BDNF expression. The results of BNDF labeling were seemingly consistent with the viewpoint of the PrL, IL, and Cg1's different levels of involvement in fear memory. During situational reminder, the Cg1 and PrL might enhance synaptic plasticity, but the IL reduces synaptic plasticity for regulating situational reminder. In other words, in the retrieval fear memory, the Cg1 and PrL neurons may be involved in the BDNF synaptic plasticity to ensure a connection with the relevant neural substrates. However, the BDNF synaptic plasticity of the IL neurons was likely weakened to connect with adjacent neurons.

### 4.2. The Amygdala and PTSD

Previous studies suggested that the amygdala regulated negative emotional responses and was involved in fear conditioning [38]. Moreover, the amygdala was shown to encode aversive and negative stimuli, and then, this negative information was associated with the contextual stimulus [39]. PTSD symptoms are present in fear and negative emotional responses, and the amygdala plays an essential role in PTSD [40]. For example, PTSD research on animals using the predator scent model showed that excitations of the BLA through high-frequency stimulations could interfere with predator scent-related anxiety and avoidance responses [41]. The inescapable footshock model of PTSD showed that animals with footshock treatments exhibited a higher expression of norepinephrine in the amygdala. In contrast, bilateral microinfusions of the beta-adrenergic receptor antagonist propranolol revealed a lower locomotive activity, indicating that the activity of the amygdala's adrenergic neurons mediated PTSD symptoms [42]. A manganese-enhanced magnetic resonance imaging study indicated that PTSD animals induced higher signals in the BLA and striatum and lower activity in the IL in a single prolonged-stress PTSD model. This indicated that the prefrontal cortex inhibited the neuronal activity of the amygdala and striatum under the single prolonged-stress procedure [43]. Furthermore, a recent single prolonged-stress procedure in a PTSD animal model found that anxiety behavior and fear memory occurred one day after the procedure and were associated with decreased activated glutamate neurons and increased activated GABA neurons in the BLA. Ten days after the procedure, the animals exhibited enhanced anxiety and impaired fear memory associated with increased glutamate and GABA transmissions in the BLA, indicating that the different PTSD stages showed a distinct pattern of glutamate and GABA neuron activities in the amygdala [44].

The present result showed that BDNF protein expression was higher in the amygdala during situational reminders. This finding supports the view that the amygdala regulates fear-related PTSD symptoms; moreover, the BDNF involvement of the amygdala in the present result has extended the previous evidence in the studies of behavioral pharmacology and magnetic resonance imaging.

### 4.3. The Hippocampus and PTSD

Accumulated evidence has shown that the hippocampus governs context-related conditioned learning [45–47], in which the contextual environment (i.e., the CS) is conditioned by a physiological or survival stimulus (i.e., the US) [48]. Because the US is a fear-related aversive and negative effect, the contextual CS has the aversive fear effect, following the aversive conditioning of the CS and US; this is termed fear conditioning [39]. Accordingly, the hippocampus does not mediate the reward or aversive valence itself; instead, the hippocampus only regulates a single and whole ensemble of contextual components [39].

On the other hand, the subareas of the hippocampus (i.e., the CA1, CA2, CA3, and DG) may play different roles in fear conditioning and fear extinction memory [23, 26, 49]. For example, under a long-term stress treatment, the CA1 of the hippocampus exhibited decreased BDNF mRNA expression and increased *TrkB* mRNA expression in the predator scent-induced stress paradigm [26], indicating that the CA1 was involved in the stress event. BDNF protein expression of the DG was lower in cue fear conditioning, and the DG plays an inhibitory role in cue fear conditioning [23]. Another study used the contextual fear conditioning paradigm and showed that lesions of the CA3 enhanced fear behavior in the acquisition stage of fear conditioning. Moreover, lesions of the CA1 and DG impaired freezing behavior in the retrieval stage of fear memory, indicating that the CA3 mediated the acquisition of fear memory and the CA1 and DG regulated the retrieval of fear memory [49].

In the present PTSD study, the CA1, CA2, CA3, and DG of the hippocampus all exhibited significantly lower BDNF expression in the third retrieval session of situational reminder. Therefore, our data are not consistent with this prior evidence. This discrepancy in evidence may be due to the different paradigms and stages of fear conditioning in PTSD. This issue should be investigated in further studies.

### 4.4. Neural Connections among the mPFC, Amygdala, and Hippocampus for PTSD during the Situational Reminder

Previous literature has reported that the mPFC, amygdala, and hippocampus were connected for playing different roles in PTSD [28, 50]. For example, a neuroimaging review suggested that during the sympathetic stage of PTSD, the mPFC and hippocampus both revealed hypoactivity associated with the hyperactivity of the amygdala [28]. This viewpoint was supported by another study, which indicated that exposing patients to severe stress produced deficits in the mPFC inhibition to suppress the hyperactivity of the amygdala [50]. For example, in severe stress, the mPFC loses its inhibitory function to the amygdala, and the deficit of the hippocampus disrupts the function of declarative memory but facilitates the nondeclarative memory of fear conditioning [50]. Another study found the mPFC and the hippocampus to be negatively connected to the amygdala, whereas the mPFC and hippocampus were positively connected [28]. In this neural relationship of the mPFC, hippocampus, and amygdala, the neural activity of the mPFC and hippocampus appeared lower, but a higher neural activity of the amygdala occurred when PTSD patients experienced a severe stress event, indicating the contribution of the neural connection in regulating PTSD symptoms [28]. However, the present data were not completely consistent with the previous viewpoint in neuroimaging research—namely, the fact that the neurons of the mPFC and hippocampus were shown to have a lower activity and the amygdala neurons exhibited a higher activity when patients experienced PTSD symptoms [28]. Our data showed that there was higher BDNF expression in the Cg1, PrL, and amygdala; however, the IL, CA1, CA2, CA3, and DG exhibited lower expression in BDNF protein tests. In our study, the PrL and IL played an opposite role in fear behavior during situational reminders, supporting the findings [33]. The data discrepancy between previous studies and ours may be due to inconsistent results from the different testing stages of PTSD and fear conditioning. In our study, we manipulated the retrieval of fear memory during situational reminder; however, the previous study conducted a fear conditioning and acquisition phase or fear extinction phase. These different testing phases may have led to the discrepancy in the results. Therefore, the acquisition, retrieval, and extinction of fear memory should be considered expressing the different patterns of neural network activity among the prefrontal cortex, amygdala, and hippocampus. Whether or not the different stages of PTSD appear in the varying neural connection patterns should be scrutinized in future studies.

### 4.5. Issue and Limitations

Some limitations should be a matter of concern. First, the issue of whether the immunohistochemical staining method was a suitable way to label BDNF proteins should be discussed. In the present study, it used immunohistochemical staining with the BDNF protein after behavioral testing. However, this method has a shortage such that the antibody of BDNF did not stain the right cellular and subcellular elements. The BDNF protein was located in the Trk receptor and surrounding postsynaptic neuronal membranes but not inside neuronal membranes [17]. BDNF is taken up by the presynaptic neurons via axonal retrograde transport into the cell body of this presynaptic neuron resulting in neuronal survival [51]. Therefore, the present data of BDNF protein expression with immunohistochemical staining have a limitation for BDNF labeling in a correct cellular element. In contrast, the western blot and ELISA approach with BDNF should be considered to prevent the deficits of the immunohistochemical staining method in further studies. Second, the small sample size for numerous Pearson correlation tests in BDNF protein and BDNF mRNA measurements should be a matter of concern because fewer data might lead to lower power values. In the neuroscience field, the small sample size (*n* = 4-6) is often used to compare the different effects between control and experimental groups with the *t*-test or *F*-test. However, the Pearson correlation test with the small sample size should be further considered for its effect size and power value.

To test BDNF protein and BDNF mRNA expression for comparing the nonfootshock and footshock groups, the PrL and IL, respectively, showed increases and decreases in the BDNF protein expression for the footshock group; however, the BDNF mRNA expression in the mPFC was lower in the footshock group. These results showed a conflict between the BDNF mRNA and BDNF protein expressions. This discrepancy in data might be due to the fact that the assessment of BDNF mRNA focused on the whole mPFC; however, the measurement of BDNF protein expression narrows down the subareas of the mPFC (i.e., PrL and IL). Thus, it shows the different functions between the PrL and IL. Furthermore, why did the PrL and IL appear to have an opposite expression in BDNF protein expression? A possible explanation is that the data of BDNF protein was consistent with the viewpoint that the PrL plays a role to enhance fear behavior, but the IL was involved in the reduction of fear behavior [52]. The present issue that whether the subareas of the mPFC contribute a different function to the fear conditioning and PTSD symptoms should be scrutinized in further studies.

### 4.6. Conclusion

In the retrieval of situational reminders, the subareas of the mPFC such as Cg1 and PrL and the amygdala showed a higher BDNF protein expression. However, the IL and the subareas of the hippocampus including CA1, CA2, CA3, and DG revealed a lower BDNF protein expression. The data of the BDNF mRNA levels were similar to those of BDNF protein expression: the levels of BDNF mRNA were higher in the mPFC and amygdala but lower in the hippocampus for the footshock group. In the neural connection analysis, we found that some connections between the mPFC, amygdala, and hippocampus changed the connection property from positive to negative. These connections included the PrL projections to the subareas of the mPFC (i.e., PrL-Cg1 and PrL–IL) and the subareas of the hippocampus (i.e., PrL-CA2, PrL-DG, and PrL-CA3), the amygdala projections (i.e., IL-amygdala, amygdala-CA2, and amygdala-PC), and the projections of the IL-PC, PC-CA3, and CA1-CA2. The reexperience of PTSD traumatic memory is seemingly to weaken the neural network of the mPFC, amygdala, and hippocampus that exhibited negative connections.

## Figures and Tables

**Table 1:** Pearson correlation tests conducted to analyze the relationship between freezing behavior and BDNF protein expression levels in selected brain areas (*n* = 10) during the final retrieval session of situational reminders.

	Cg1	PrL	IL	CA1	CA2	CA3	DG	Amygdala	PC
*r*	0.72	0.76	-0.61	-0.76	-0.75	-0.83	-0.60	0.75	-0.49
*p*	<0.05^∗^	<0.05^∗^	ns	<0.05^∗^	<0.05^∗^	<0.05^∗^	ns	<0.05^∗^	ns

**Table 2:** Pearson correlation tests conducted to analyze the relationship between freezing behavior and BDNF mRNA levels in selected brain areas (*n* = 12) during the final retrieval session of situational reminders.

	mPFC	Amygdala	Hippocampus	PC
*r*	0.80	0.84	-0.62	-0.28
*p*	<0.05^∗^	<0.05^∗^	<0.05^∗^	ns

## Data Availability

The raw data can be accessed through the following link: https://www.dropbox.com/sh/ao9rnafpinp6qxb/AADqYPNwXL1rXWv5ehUKdfvha?dl=0.
